# Antiglaucoma pharmacotherapy

**Published:** 2012-09-25

**Authors:** CP Tătaru, VL Purcărea

**Affiliations:** * Ophthalmology Department, “Carol Davila” University of Medicine and Pharmacy, Bucharest; ** ”Carol Davila” University of Medicine and Pharmacy, Bucharest

**Keywords:** Glaucoma, Monotherapy, Fixed combinations, Neuroprotection

## Abstract

This review presents the pharmacotherapeutic approaches available in the treatment of glaucomatous optic neuropathy. Although its etiology is multi-factorial, currently, the main therapy is to decrease intraocular pressure. New therapies are being developed; the current trend is a retinal ganglion cell neuroprotection. Neuroprotection is achieved by combining antihypertensive agents with drugs that directly protect the optic nerve by promoting cell survival and inhibition of neuronal signals that initiate apoptosis. The treatment should also preserve the ocular hemodynamics, ensure proper patient compliance and be free of side effects.

Glaucoma is a group of eye diseases, characterized by progressive and irreversible structural and functional degradation of the optic nerve, with the potential to cause blindness. The specific changes consist of a loss of optic nerve substance, in terms of excavation and glaucomatous visual field defects of varying severity [**16**]. The irreversible loss of the visual function in the pathophysiology of this disease has made glaucomatous optic neuropathy be intensively studied, in order to identify the mechanisms of occurrence and the ways to suppress them. Being the first worldwide cause of irreversible blindness [**21**], glaucoma occurs unexpectedly, often being discovered in an advanced state due to the absence of symptoms or to insidious symptoms that develop very slowly in time. Thus, we are facing the fact that nearly half of those suffering from glaucoma are unaware of having the disease [**5**].

The etiology of glaucomatous optic neuropathy includes a wide variety of factors, resulting in loss of retinal ganglion cells and their axons as shown by specific morphological changes of the optic nerve during the fundus examination. The functional correspondent of the ganglion cell and axon loss is shown by specific and irreversible defects of the visual field.

The initial damage in glaucoma is not fully known in terms of pathophysiology. It can occur both in the retinal ganglion cell body and in the axons. Whatever the initial injury or molecular mechanisms of glaucomatous optic neuropathy, the consequence is death of the ganglion cell and its axon. Increased intraocular pressure is the most important risk factor for the development of glaucoma, but not the only one. Physiological studies have shown that there are many other factors involved in ganglion cell death and the diagnosis of glaucoma can now be made even if the patient does not have intraocular hypertension [**19**]. The other risk factors for glaucoma are the following: age over 60, African and Asian race, myopia forte, vascular hypotension, vasospasm, diabetes mellitus, cortisone medication, positive family history of glaucoma, etc. [**5**].

According to the pressure theory in the hypertensive glaucoma, ocular hypertension and vascular circulatory dysfunction are mechanisms that produce the early injury in glaucomatous optic atrophy. Initially, there would be an obstruction of axoplasmic flow in the retinal ganglion cell axons at the lamina cribrosa level, optic nerve microcirculation perturbation in the lamina cribrosa and disturbance of glial and connective tissue of lamina cribrosa. Then, the neurons injured by the excess production of free radicals such as nitric oxide, would release glutamate, which has a toxic metabolic effect. Excess glutamate is particularly associated with the development of glaucoma. Another pathogenic theory of initial lesions in glaucoma neuropathy involves cell apoptosis. 

Glaucomatous excavation appears because of the loss of retinal ganglion cell axons, which is accompanied by the loss of their cell body. It turned out that apoptosis (or programmed cell death) is a primary retinal ganglion cells destruction mechanism in glaucoma, being involved in all stages of the disease. Apoptosis consists in the destruction of the cell nucleus, cell membrane rupture and phagocytosis of the disorganized cell by the neighboring cells.

Although ocular hypertension has an important role in the pathogenesis of glaucoma, there are patients in whom its therapeutic control is not enough to stop the progression of the disease. Glaucomatous optic nerve changes are also seen in patients with normal or low intraocular pressure, like in normal pressure glaucoma. This means that there are other factors involved in the initiation and development of glaucoma [**9**].

Vascular factors (especially vascular insufficiency) are involved in the etiopathogenesis of glaucoma. There are many associations between vascular circulatory disorders and glaucoma. Clinically, vascular risk factors should be sought mainly in patients with normal intraocular pressure who present progressive visual field defects.

The therapeutic aim in glaucoma is to prevent or modify the risk factors, especially the intraocular hypertension by using hypotensive medication. The other two main therapeutic directions are vascular therapy and neuroprotection therapy [**17**].

Topical medication, laser therapy and surgery are the main therapeutic approaches in glaucoma.

Hypotensive antiglaucoma agents are classified according to the pharmacological category [**7**]:

1)-adrenergic agonists for topical use: a) dipivefrin 0.1%, epinephrine 0.25-2% b) selective alpha 2: apraclonidine 0.5-1% (Iopidine), Brimonidine 0.2% (Alphagan).

2)-adrenergic antagonists for topical use: a) selective beta1: betaxolol 0.25-0.5% (Betoptic, Betoptic S), b) non-selective: timolol 0.1, 0.25, 0.50% (Timolol, Timoptic, Timabak, Timogel, Nyolol) c) non-selective with intrinsic sympathomimetic activity: carteolol 2% (Carteol, Fortinol, Carteabak), d) non selective-Levobunolol [3].

3) carbonic anhydrase inhibitors: a) topical: 1% brinzolamide (Azopt), dorsolamidă 2% (Trusopt), b) systemic: acetazolamide (acetazolamide, Diamox).

4) cholinergic agents (parasympathomimetics) - topical: a) direct action: pilocarpine 0.5-4% (Pilocarpine, Isopto Carpini, Pilogel), aceclidine 2% (Glaucostat, Glauconorm), carbacholina 0.75-3% (Isopto Carbachol), acetilcholina 1% (Miochol) b) indirect action: demecarium bromide 0125, 0.25% (Humorsol).

5) prostamides and derived prostaglandins for topical use: a) prostamides: 0.03% bimatoprost (Lumigan), b) prostaglandin analogues: latanoprost 0.005% (Xalatan), travoprost 0.004% (Travatan), tafluprost 0.0015% (Taflotan) c ) docosanoide: unoprostona 0.12, 0.15% (Rescula).

6) osmotic agents for systemic use to control acute increases in intraocular pressure: glycerol 1-5 g/kg body p.o, mannitol intravenously 1-1.5 g/kg body. 

7) combinations of antiglaucoma agents for topical use-a) 0.5% timolol and bimatoprost 0.03% (Ganfort) b) timolol 0.5% and latanoprost 0.005% (Xalcom) c) timolol 0.5% and travoprost 0.004% (DuoTrav) d ) timolol 0.5% and Brimonidine 0.2% (Combigan), e) timolol 0.5% and dorzolamide 2% (Cosopt), f) timolol 0.5% and pilocarpine 2% (Fotil), carteolol 2% and pilocarpine 2% (Carpilo).

When monotherapy proves insufficient in controlling intraocular pressure, drug combinations are used. The additive effect appears when the drugs used have different mechanisms of action. In addition, the compliance is higher and there are fewer side effects.

The main mechanisms of action of antiglaucoma therapeutic agents are: reduced production of aqueous humor from the ciliary body, increased evacuation of the aqueous humor through the trabecular meshwork and through the uveoscleral way.

In terms of pharmacokinetics, the bioavailability at receptor level in the ocular tissues of the antiglaucoma topical agents is influenced by:

1) The kinetics of the substance in the conjunctival sac.

 After topical administration, the substance is mixed with tears in the conjunctival sac, and thus a substantial part of it is lost through the lacrimal drainage system, a small amount mixes with the precornean tear film and penetrates the cornea. The degree of saturation of the precornean tear film influences the amount of substance that penetrates the cornea and so the biodisponibility of the substance. In order to prolong the retention time of the substance in the conjunctival sac and its increased saturation into the precornean tear film, there have been developed vehicles for topical antiglaucoma substances: suspensions and emulsions, soluble polymers (methylcellulose), ointments, soluble gels (high viscosity).

2) Corneal Penetrability

The penetration of antiglaucomatous agents through the cornea complies with the concept of differential solubility, which means that only substances that are both lipid-soluble and water-soluble can penetrate the healthy cornea (the cornea having a lipo-hydro-lipidic structure).

3) Distribution and clearance of substance in the eye.

These features are detailed while focusing on some of our antiglaucoma agents.

1) Adrenergic agonists

a)Apraclonidine (Iopidine)- has a para amino structure and is derived from clonidine. It decreases aqueous humor production and increases its elimination from the trabeculum by reducing pressure in the episcleral veins. Long-term administration leads to tachyphylaxis, so it is recommended only in acute cases and post laser therapy. 1 hour after administration, it lowers the intraocular pressure by 20%. The maximum effect occurs after 4-5 hours and lasts for 12 hours. It has an additive effect with other antiglaucoma agents and its increased polarity limits the penetration through the blood-brain barrier, reducing adverse effects on the central nervous system [**11**].

b)Brimonidine (Alphagan)- is a potent antiglaucoma agent used as first-line antihypertensive therapy. It is recommended mainly for chronic use in patients with concomitant cardiopulmonary disease or contraindication to beta-blockers. It reduces IOP by decreasing aqueous humor production and increasing uveoscleral outflow, the effect lasting up to 12 hours. Its high selectivity is accompanied by a lack of local adverse reactions. It has a good retinal availability and neuroprotective effect on experimental models.

2)Adrenergic antagonists

a)Timolol- is one of the most popular and prescribed antiglaucoma agents, as a first-line drug in most forms of open angle glaucoma and ocular hypertension [**2**]. It reduces the production of aqueous humor by acting on the ciliary body, inhibiting beta 1 and beta 2 adrenergic receptors. The duration of action is of more than 8 hours. Effects may be lost in time due to the long-term use. In addition, it can be associated with local and systemic side effects such as bradycardia, bronchospasm, hypoglycemia, depression, dry eye. Timolol maleate (Timoptic XE) is a pharmaceutical improved formula that uses an anionic polysaccharide derivative of gellan gum as a vehicle. In contact with the cations from the tear film, it transforms into a gel that persists longer in contact with the eye. It is prescribed only once a day, increasing patient compliance [**18,20**].

b)Carteol- is an antiglaucoma agent with a very high efficiency. The half-life of its 8-hidroxicarteolol metabolite is twice as long as the basic substance; hence, the higher bioavailability and increased duration of action compared with other beta adrenergic antagonists. It is used in all forms of glaucoma, is well tolerated and is preferred in patients with hypercholesterolemia, as it modifies the lipid curve [**15,16**]. 

c)Betaxol- is the first beta 1 cardio selective adrenoceptor antagonist used in ophthalmology. Its ocular hypotensive action is lower, but the side effects, especially the cardiopulmonary ones, in the elderly, are reduced [**6**]. It has proven to have a neuroprotective action. 

3) Carbonic anhydrase inhibitors

Dorzolamid (Trusopt) is a chemically non-bacteriostatic derivative of sulfonamide, which decreases the production of aqueous humor. After corneal penetration, it inhibits the carbonic anhydrase from the ciliary body, slowing local bicarbonate production and so decreasing sodium and fluid transport, thus reducing aqueous humor secretion [**13**]. It can be used in combination with beta-blockers. It is a second line agent in open-angle glaucoma and ocular hypertension, preferably in patients with contraindication to beta-blockers. It is administered three times a day. In patients with compromised corneal endothelium it can cause irreversible corneal edema, that is why it is used with prudence in the management of the glaucoma attack with corneal edema – in that case we prefer a carbonic anhydrase inhibitor in the systemic administration. Brinzolamide (Azopt) is a drug with a good hypotensive effect, similar to dorzolamide and it is prescribed twice a day [**14**].

4)Cholinergic agents

Pilocarpine - is a miotic agent used in closed or narrow angle glaucoma. Through its miotic action, it suppresses the papillary block. Local side effects are significant, affecting the patient’s quality of life. Therefore, pilocarpine was intensely studied and several administration forms were developed, aiming to achieve the pharmacological action of the active substance with the minimum concentration possible and a prolonged action time using rare instillations for fewer side effects [**10**]. A) methylcellulose soluble polymers: increased retention of pilocarpine in the conjunctival sac and corneal penetrability; B) pilocarpine gel: contains a high viscosity acrylic vehicle that allows a 24 hours effect; C) administration systems controlled by membranes- the system is an insert in the conjunctival sac, where pilocarpine is released gradually at a rate of 20 or 40 micrograms per hour, being active for 7 days; D) soft contact lenses soaked in pilocarpine; E) transdermal delivery system - is a system consisting of a pilocarpine skin patch applied supraclavicularly; F) electronic alarm system – the compliance is significantly increased, G) pilocarpine encapsulated in liposomes - has an extended effect in the experimental studies.

5) Prostaglandin derivates

Latanoprost (Xalatan) - available as a pro-drug, is a derivative of PGF2 alpha. This pro-drug is activated by enzymatic hydrolysis in the cornea. The pharmacological response is mediated by prostanoid receptors. The uveoscleral outflow is increased and its action lasts for 24 hours. The most common adverse reactions are conjunctival hyperemia and iris and eyelashes pigmentation [**22**]. Like all derivatives of prostaglandins and prostamides, Xalatan can be used as first line therapy in open angle glaucoma, as monotherapy or in combination with beta-blockers. Travoprost and bimatoprost have a hypotensive action comparable to latanoprost (20-30% decrease IOP by increasing uveoscleral outflow), all being administered once a day [**11,1,4**]. This class of compounds is more often used as first-line therapy in glaucoma, according to the therapeutic guidelines [**5**].

New therapies of glaucoma

There are a number of new antiglaucoma pharmacological agents that are currently in clinical trials, such as the following examples: 

a) phenoxyacetic acid derivatives – ethacrynic acid: appears to be a promising substance in the treatment of glaucoma, markedly reducing the intraocular pressure after topical or intracameraly administration. It increases aqueous humor elimination in experimental studies by changing the shape and altering cellular microtubules through its sulfhydryl group reactivity.

b) steroid antagonists – mifepristone and its derivatives: show to be promising in the treatment of glaucoma, although the mechanism of ocular hypotensive effect is still unclear.

c) atrial natriuretic peptide - its hypotensive action has been experimentally proven, the anterior uvea containing the peptide and its receptors. Intraocular pressure reduction is achieved by reducing aqueous humor secretion directly from the ciliary body.

d) angiotensin converting enzyme inhibitors - they could play a role in the process of decreasing the production of aqueous humor and controlling retinal blood flow through the renin-angiotensin system present in the eye tissues. The angiotensin II receptors of the retinal blood vessels may play an important role in the autoregulation of blood flow in the retinal and optic nerve head.

e) the group of ocular hypotensive lipids: is a possible new class of antiglaucoma agents, which replaces the carboxylic acid group of PGF2 alpha with a neutral group. Unlike latanoprost, metabolic compounds are stable and do not interact with prostanoid receptors. In clinical trials, the hypotensive effect was significant, lasting for 24 hours and the side effects were reduced. The safety profile was very good, this class showing very promising long-term therapy for open angle glaucoma.

e) neuroprotective agents: they are given a major importance in glaucoma therapy. These substances may interfere with the mechanisms of retinal ganglion cell apoptosis and they provide effective neuroprotection [**12**]. These substances include Brimonidine, latanoprost, ginkgo biloba derivatives, N-methyl-D-aspartate antagonists (memantine), calcium channel blockers (lomarizina), glutamate antagonists (Riluzole), nitric oxide synthase inhibitors [**8**], neurotrophic factors, substances that reduce free radicals, melatonin, neuroprotective vaccines, erythropoietin, caspase inhibitors, nipradilol.

In conclusion, it can be said that currently, there is a variety of hypotensive antiglaucoma agents from where a customized therapy, monotherapy, as well as drug combinations can be chosen for each patient. However, future therapies in glaucoma will focus not only on lowering the IOP but also on offering an effective neuroprotection, redefining success in terms of effective conservation of vision or visual field deficits reversibility. New therapies should also preserve ocular hemodynamics, provide good patient compliance and be free of side effects. Efficient neuroprotection might be achieved by combining antihypertensive agents with those that directly protect the optic nerve by promoting cell survival and by inhibiting neuronal apoptosis initiating signals [**19**]. For assessing the effectiveness of neuroprotective agents, it is necessary to develop new methods that detect the neuronal damage of the optic nerve before the onset of visual field deficits.

The regeneration of CNS axons by molecular genetics and cellular techniques is an interesting research topic and may enable the recovery of lost fibers in the glaucomatous optic nerve atrophy. The synthesis of cytokines and growth factors for astrocytes, as well as the alteration of adhesion molecules expression on the cell surface (such as neural adhesion molecule) might be exciting future therapeutic modalities. 
